# Nitrogen and Sulphur Relations in Effecting Yield and Quality of Cereals and Oilseed Crops

**DOI:** 10.1100/tsw.2001.381

**Published:** 2001-12-11

**Authors:** B.K. Nad, T.J. Purakayastha, D.V. Singh

**Affiliations:** Division of Soil and Agricultural Chemistry, Indian Agricultural Research Institute, New Delhi, India

**Keywords:** N&S, mustard crop, wheat crop, rice crop, seed yield, oil yield, N uptake, S uptake, N sources, S sources, N:S ratio, nutrient mobility (S)

## Abstract

Nitrogen and sulphur, both vital structural elements, are especially needed for the synthesis of proteins and oils. Investigations revealed the required application of sulphur is one half to one third the amount of nitrogen, and the ratio becomes narrower in mustard (Brassica juncea L.), followed by wheat and rice. The efficiency of an increased level of nitrogen required a proportionately higher amount of sulphur. A critical investigation on the effective utilization of applied vis-à-vis absorbed nitrogen in wheat and mustard envisaged accumulation of NO_3_-N in vegetative parts when sulphur remained proportionately low. Application of sulphur hastened the chemical reduction of absorbed NO_3_ for its effective utilization. The effect was more pronounced in mustard than in wheat. Easily available forms of sulphur, like ammonium sulphate and gypsum, as compared to pyrite or elemental sulphur, maintained adequate N to S ratio in rice, resulting in a reduction in the percent of unfilled grain, a major consideration in rice yield. A narrow N to S ratio, with both at higher levels, increased the oil content but raised the saponification value of the oil, a measure of free fatty acids. Whereas, a proportionately narrow N to S ratio at moderate dose resulted in adequately higher seed and oil yield with relatively low saponification value, associated with increased iodine value of the oil, indicating respectively low free fatty acids and higher proportion of unsaturated fatty acids, an index for better quality of the oil.

## INTRODUCTION

As far as plant nutrition is concerned, no individual nutrient element may bring in the overall physiological and metabolic activities effecting the growth and yield in plants. With the growing demands for additional applications of sulphur in intensive cultivation systems, maintenance of a balanced supply of sulphur with specific relations to the amount of nitrogen has become inevitable, in view of the soils and cropping systems, in sustaining both yield and produce of the crops. Both nitrogen and sulphur play vital roles as structural elements in plants and, more importantly, as constituents of proteins and oils. Further, nitrogen metabolism is greatly influenced by sulphur. At inadequate uptake of sulphur, synthesis of proteins and oils is suppressed, and consequently, the absorbed nitrate that is accumulated as nonprotein elements may result not only in loss of yield, but also may impair the quality of crop produce.

Efforts have been made to consolidate a report in this regard based on various investigations both in field and net house conditions on cereals and oilseed crops using isotopic sulphur ($^{35}S$) as per necessity.

## N AND S RELATIONS IN EFFECTING GRAIN YIELD OF CEREAL AND OILSEEDS

In order to assess N–S relations in crops, utilization efficiency of applied sulphate in wheat was studied in a pot culture experiment with increasing levels of nitrogen. The crop was grown to maturity in 4-kg pots with processed low-fertility soil (Typic Ustochrept). The treatments consisted of increased ratios of applied N to S, i.e., 0:1, 1:1, 2:1, 3:1, and 4:1 (0, 60, 120, 180, 240 kg N $ha^{-1}$ and 60 kg S $ha^{-1}$, respectively). The source of sulphur was labelled with $^{35}S$. The crop received 90 kg $P_2O_5$ and 60 kg $K_2O$ $ha^{-1}$ as basal.

The grain and straw produced by the treatments clearly indicated a significant influence of applied sulphur in increasing the amounts of both grain and straw at $N_{120} + S_{60}$ (i.e., N to S ratio 2:1), as compared to no sulphur application ($N_{120}$). With a further increase in nitrogen level but the same level of sulphur at 60 kg $ha^{-1}$ (i.e., >2:1), there was no increase in either grain or straw; rather a downward trend was recorded at a 4:1 ratio of N to S (Table 1).

A radiochemical study revealed a steady rise in uptake of fertilizer-S as well as total S uptake in both grain and straw of the crop, due to increased ratios of applied N and S. The percentage of sulphur derived from fertilizer (percent Sdff) increased sharply at 1:1 level of N to S, as compared to that of 0:1 level in both grain and straw. Percent utilization of the applied S also followed a similar trend to that of percent Sdff. Both values continued to increase, though to a lesser magnitude, with the increments of the N levels and attained a maximum at a 4:1 level of N to S — that is, at 60 kg S $ha^{-1}$ superimposed with 240 kg N $ha^{-1}$.

In order to appraise the response to sulphur as such in cereal and oilseed crops, the study was initiated in greenhouse conditions with the application of $^{35}S$-labelled sulphate. The observations showed a clear difference in the rate of increase in dry matter production and uptake of sulphur by wheat and mustard. While there was a higher percentage of increase in dry matter of wheat, the rate of uptake of S recorded in mustard was much more than in wheat. This was corroborated to a great extent with both wheat and rice against mustard in the detailed study under field conditions. Both direct and residual effects of applied sulphur were also assessed in different sequences of three cereal-oilseed crops, namely, rice-wheat, wheat-rice, rice-mustard, and mustard-rice (Table 2).

**TABLE 1**
**Grain and Straw Production by Wheat with the Application of N and S in Increasingly Wider Ratios**

| | Yield (g $pot^{-1}$) | | | |
|---|---|---|---|---|
| | Grain | | Straw | |
| Treatment* | Without S | With S | Without S | With S |
| $N_0$ | 4.7 | 5.0 | 5.6 | 6.0 |
| $N_{60}$ | 9.4 | 10.2 | 11.3 | 12.1 |
| $N_{120}$ | 11.5 | 13.9 | 13.4 | 15.9 |
| $N_{180}$ | 16.8 | 16.7 | 19.0 | 19.1 |
| $N_{240}$ | 18.4 | 16.6 | 22.5 | 20.2 |
| CD at 5% | 2.1 | 2.3 | 2.9 | 2.8 |

* Subscripts of 0 to 240 with N are 0 to 240 kg N $ha^{-1}$; CD = critical difference.

**TABLE 2**
**Mean Yield of Rice, Wheat, and Mustard Grown in Different Sequences (q $ha^{-1}$)**

| S level* | Rice–Wheat | | Wheat–Rice | | Rice–Mustard | | Mustard–Rice | |
|---|---|---|---|---|---|---|---|---|
| (kg $ha^{-1}$) | Rice | Wheat | Wheat | Rice | Rice | Mustard | Mustard | Rice |
| $S_0$ | 27.2 | 36.1 | 30.0 | 28.8 | 27.7 | 6.67 | 6.22 | 24.3 |
| $S_{20}$ | 27.3 | 36.3 | 30.8 | 28.6 | 28.3 | 7.07 | 7.22 | 25.9 |
| $S_{40}$ | 29.6 | 38.2 | 33.2 | 28.9 | 29.6 | 7.00 | 7.47 | 27.3 |
| $S_{60}$ | 31.7 | 38.3 | 35.3 | 29.3 | 32.7 | 7.33 | 7.47 | 28.3 |
| $S_{80}$ | 32.9 | 40.0 | 38.8 | 30.9 | 34.3 | 7.20 | 7.33 | 28.1 |
| Average | 29.7 | 37.8 | 33.6 | 29.5 | 30.5 | 7.05 | 7.14 | 26.6 |
| CD at 5% | 0.7 | 0.9 | 0.9 | NS | 0.7 | NS | NS | 1.0 |

* Subscripts of 0 to 80 with S are 0 to 80 kg S $ha^{-1}$; CD = critical difference.

By and large, there was an increase in yield, in the order of wheat followed by rice and mustard, due to S application, with S composition being higher and enhanced with its application in mustard at the recommended NPK basal level. Though there appeared to be a residual effect of applied sulphur on rice, the overall individual yield of wheat was greater when grown as a residual crop after rice than as a direct one. Yield of mustard, however, was not much affected by the order of the sequence (Table 2). Nevertheless, the higher percent utilization of applied S in mustard, as observed in greenhouse study, may likely be better utilized for its oil content, whereas a balancing of the overall nutrients could affect the yield of cereals directly.

## MOBILITY OF SULPHUR AS INFLUENCED BY NITROGEN

In assessing the nitrogen and sulphur relations as plant nutrients, sulphur mobility was studied in mustard plants, as influenced by concentration of nitrogen, using the tracer technique. The computed values for mobility of a constant level of $^{35}$S-labeled sulphur against the increasing concentration of nitrogen in solution were significantly influenced and increased up to the level of 300 ppm N. The value was almost stable up to 400 ppm N, but it decreased significantly when the level of nitrogen was increased to 600 ppm (Table 3).

Accordingly, the percent mobility of added sulphur ranged from 11.03% when no nitrogen was in the solution to 22.29% with 300 ppm N, but decreased to 16.2% when the level of N was further increased to 600 ppm.

## GROWTH PARAMETERS OF RICE AS INFLUENCED BY SOURCES OF SULPHUR

A pot culture experiment was conducted to study some of the growth parameters and nutrient uptake in rice, when grown in a Typic Ustochrept soil with the application of sulphur as elemental sulphur, gypsum, pyrite, and ammonium sulphate. The treatments received 120 kg N, 60 kg $P_2O_5$, and 30 kg $K_2O$ ha$^{-1}$ from sulphur-free materials as basal.

Grain and dry matter yield of rice showed the largest increase due to ammonium sulphate, followed by pyrite and gypsum, but least due to elemental sulphur. There was an appreciable effect on the percentage of unfilled grain because of the treatments. Application of ammonium sulphate, closely followed by gypsum and pyrite, reduced the percentage of unfilled grain (Table 4). This was also reflected in the grain to straw ratio. Ammonium sulphate proved to be most effective in the uptake of sulphur in grain, followed by gypsum and pyrite. The change in uptake of sulphur closely resembled that of P, Mg, and K (data not shown). Percent Pdff was also in conformity with its uptake. It is evident from the results that the problem of unfilled grain in rice may well be countered through balanced nutrition, especially that of sulphur *vis-à-vis* phosphorus, whereas, Ca or Fe may have an indirect effect on filling the grains.

**TABLE 3**
**Mobility of Sulphur in Mustard as Affected by Levels of N Applied**

| N level | S mobility (μg S per g dry wt) | % S mobility |
|---|---|---|
| $N_0$ | 9.9 | 11.0 |
| $N_{50}$ | 11.6 | 12.8 |
| $N_{100}$ | 14.3 | 15.9 |
| $N_{200}$ | 17.3 | 19.2 |
| $N_{300}$ | 20.1 | 22.3 |
| $N_{400}$ | 20.8 | 23.2 |
| $N_{600}$ | 14.6 | 16.2 |
| CD at 5% | 1.2 | 1.4 |

* Subscripts of 0 to 600 with N are ppm N.

## NITRATE-NITROGEN METABOLISM IN WHEAT AND MUSTARD CROP

A pot culture experiment was conducted with mustard (*Brassica juncea* L.) cv. Pusa Bold and wheat (*Triticum aestivum* L.) cv. HD-2307, as test crops in a Typic Ustochrept soil from IARI farm, New Delhi, involving selected combinations of 0, 30, 60, and 90 kg S ha$^{-1}$ and 0, 30, and 60 kg MgO ha$^{-1}$ and 1 ppm Mo. The results of the experiment revealed that the concentration of $NO_3$-N in both wheat and mustard straw was higher when only N, P, and K were applied as basal. The application of S, Mg, and Mo, or their combinations generally reduced $NO_3$-N accumulation[1]. The increased levels of S reduced the $NO_3$-N more in wheat straw than in mustard. The reduction in $NO_3$-N in straw resulted in an increase in N content and thereby protein yields in grain of both the crops. The highest protein yield was recorded to be 914.4 mg pot$^{-1}$ with $S_{60}$ $Mo_1$, against 676.7 mg pot$^{-1}$ in control in wheat, whereas, the values were 875.5 mg pot$^{-1}$ with $S_{90}Mg_{60}Mo_1$, against 563.1 mg pot$^{-1}$ in control in mustard. It is apparent from the values of total uptake of nitrogen (data not presented) that wheat has higher demand for N as compared to mustard. Thus it required correspondingly higher amounts of sulphur to metabolize $NO_3$-N in effecting protein yield, while mustard required a relatively smaller amount, since it is also reported to have a greater extracting capacity for sulphur[2]. Because of higher demand and uptake of Mg in mustard the combined effect of S, Mg, and Mo resulted in maximum reduction in $NO_3$-N in straw[3].

## MUSTARD OIL YIELD AND QUALITY OF OIL

The increase in seed yield due to higher nitrogen uptake resulted in more protein yield in mustard (*B. juncea* L.), but with further uptake of sulphur, the increase in seed yield resulted in more oil yield than protein yield. Interestingly, increase in the availability

**TABLE 4**
**Some Growth Parameters of Rice Grain as Influenced by Levels and Sources of Sulphur**

| Source and Levels of Sulphur (kg S ha$^{-1}$) | Grain Produced (g pot$^{-1}$) | % Unfilled Grain | Grain/Straw Ratio | Uptake of Sulphur (mg pot$^{-1}$) | % Pdff |
|---|---|---|---|---|---|
| Control (S0) | 12.5 | 20.4 | 1.04 | 34.7 | 31.7 |
| Amm. Sulphate | | | | | |
| 20 | 13.2 | 14.5 | 1.18 | 35.8 | 36.3 |
| 40 | 14.9 | 12.9 | 1.20 | 43.9 | 38.9 |
| 80 | 15.7 | 11.1 | 1.21 | 45.0 | 43.2 |
| Elemental S | | | | | |
| 20 | 12.8 | 20.1 | 1.05 | 36.1 | 33.1 |
| 40 | 12.7 | 18.9 | 1.05 | 35.4 | 33.6 |
| 80 | 13.0 | 18.0 | 1.08 | 37.3 | 35.8 |
| Gypsum | | | | | |
| 20 | 13.2 | 14.9 | 1.20 | 39.0 | 35.4 |
| 40 | 14.1 | 12.5 | 1.20 | 42.6 | 39.3 |
| 80 | 13.7 | 11.7 | 1.22 | 43.4 | 39.7 |
| Pyrite | | | | | |
| 20 | 13.0 | 19.1 | 1.10 | 40.4 | 32.4 |
| 40 | 13.5 | 16.7 | 1.07 | 41.8 | 35.2 |
| 80 | 13.9 | 13.9 | 1.15 | 43.0 | 40.8 |
| CD at 5% | 0.6 | 1.8 | 0.06 | 2.1 | 2.0 |

*Note:* CD = Critical difference.

**TABLE 5**
**Seed and Oil Yield of Mustard and Some Quality Parameters**

| Treatment | Seed Yield (q ha$^{-1}$) | Protein Yield (q ha$^{-1}$) | Oil Yield (q ha$^{-1}$) | Seed N:S Ratio | Straw N:S Ratio | Saponi-fication Value | Iodine Value |
|---|---|---|---|---|---|---|---|
| Control | 24.4 | 3.8 | 9.4 | 1.84 | 1.02 | 174.8 | 94.0 |
| N | 29.4 | 4.7 | 11.0 | 1.95 | 1.30 | 174.3 | 108.1 |
| NPK | 32.9 | 5.2 | 12.5 | 1.88 | 1.25 | 182.2 | 93.1 |
| NPK + S | 34.4 | 5.5 | 13.5 | 1.88 | 1.07 | 182.9 | 87.8 |
| NPK + S + Mg + Ca + FYM | 37.2 | 5.9 | 14.2 | 1.89 | 1.04 | 182.4 | 96.0 |
| CD at 5% | 2.1 | 0.4 | 0.9 | NS | 0.24 | — | — |

*Note:* CD = Critical difference.

and uptake of sulphur, to a limit, increased the oil yield, but the quality of oil was significantly and adversely affected. The saponification value, as a measure of free fatty acids, was increased with the increase in oil yield. Whereas, limited availability and uptake of sulphur, though it restricted the increase of oil yield, significantly raised the iodine value of the oil, indicating clearly the higher proportion of unsaturated fatty acids as a better quality index of the oil (Table 5).

Higher uptake of nitrogen, on the other hand, though it produced more seed yield, resulted in reduced oil yield, yet markedly improved the quality of oil by suppressing the saponification value, with a simultaneous increase in iodine value.

## CONCLUSION

The various results, as highlighted above on the aspects of growth and quality parameters, including utilization efficiency of some nutrient elements, reveal that nitrogen, a vital major plant nutrient element, depends on sulphur, among other important nutrient elements including phosphorus. Application of sulphur is inevitable, particularly when nitrogen application is raised for higher production. A proportion of sulphur to nitrogen of 1:2 to 1:3, depending upon the oilseed or cereal crops, is likely to boost and sustain the yield as well as quality of crops, especially that of mustard oil, in terms of the free fatty acid contents and proportion of saturated to unsaturated fatty acids.

| | | |
|---|---|---|
| Received: | July | 28, 2001 |
| Revised: | November | 9, 2001 |
| Accepted: | November | 10, 2001 |
| Published: | December | 11, 2001 |
